# Low-dose X-ray enhanced tumor accumulation of theranostic nanoparticles for high-performance bimodal imaging-guided photothermal therapy

**DOI:** 10.1186/s12951-021-00875-8

**Published:** 2021-05-26

**Authors:** Qiaolin Wei, Jian He, Shuaifei Wang, Shiyuan Hua, Yuchen Qi, Fangyuan Li, Daishun Ling, Min Zhou

**Affiliations:** 1 Eye Center, The Second Affiliated Hospital, Zhejiang University School of Medicine, Hangzhou, 310009 China; 2 Institute of Translational Medicine, Zhejiang University, Hangzhou, 310029 China; 3 Institute of Pharmaceutics, College of Pharmaceutical Sciences, Zhejiang University, Hangzhou, 310058 China; 4 State Key Laboratory of Modern Optical Instrumentations, Zhejiang University, Hangzhou, 310058 China; 5 Institute of Pharmacy, School of Medicine, Hangzhou Normal University, Hangzhou, 311121 China; 6 Frontiers Science Center for Transformative Molecules, School of Chemistry and Chemical Engineering, National Center of Translational Medicine, Shanghai Jiao Tong University, Shanghai, 200240 China

**Keywords:** Gold Nanoparticles, X-ray Irradiation, Raman Imaging, Photoacoustic Imaging Photothermal Therapy

## Abstract

**Background:**

Theranostic nanoparticles (NPs) have achieved rapid development owing to their capacity for personalized multimodal diagnostic imaging and antitumor therapy. However, the efficient delivery and bulk accumulation of NPs in tumors are still the decisive factors in improving therapeutic effect. It is urgent to seek other methods to alters tumor microenvironment (like vascular permeability and density) for enhancing the efficiency of nanoparticles delivery and accumulation at the tumor site.

**Methods:**

Herein, we developed a Raman-tagged hollow gold nanoparticle (termed as HAuNP@DTTC) with surface-enhanced Raman scattering (SERS) property, which could be accumulated efficiently in tumor site with the pre-irradiation of low-dose (3 Gy) X-ray and then exerted highly antitumor effect in breast cancer model.

**Results:**

The tumor growth inhibition (TGI) of HAuNP@DTTC-induced photothermal therapy (PTT) was increased from 60% for PTT only to 97%, and the lethal distant metastasis of 4T1 breast cancer (such as lung and liver) were effectively inhibited under the X-ray-assisted PTT treatment. Moreover, with the strong absorbance induced by localized surface plasmon resonance in near-infrared (NIR) region, the signals of Raman/photoacoustic (PA) imaging in tumor was also significantly enhanced after the administration of HAuNP@DTTC, indicating it could be used as the Raman/PA imaging and photothermal agent simultaneously under 808 nm laser irradiation.

**Conclusions:**

Our studied of the as-prepared HAuNP@DTTC integrated the Raman/PA imaging and PTT functions into the single platform, and showed the good prospects for clinical applications especially with the low-dose X-ray irradiation as an adjuvant, which will be a productive strategy for enhancing drug delivery and accumulation in tumor theranostics.

**Graphic Abstract:**

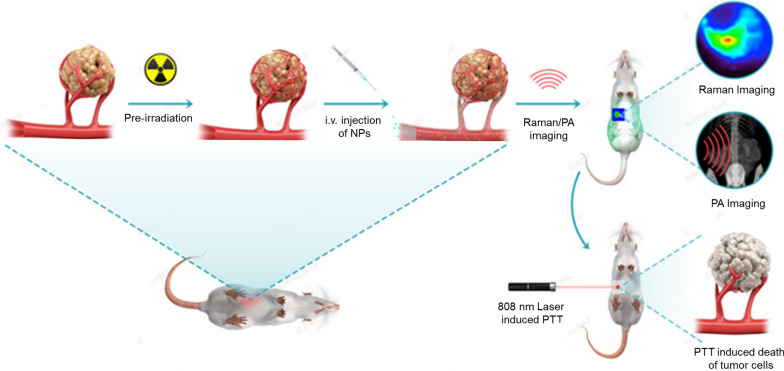

**Supplementary Information:**

The online version contains supplementary material available at 10.1186/s12951-021-00875-8. Supplementary data to this article can be found online including Materials, Supplementary Experimental Methods of in vitro and in vivo.

## Background

In recent years, the development of theranostic nanoparticles (NPs), which integrate diagnostic and therapeutic functions into a single-nanometer-sized platform, has been recognized as one of the promising strategies in the nanomedicine field [1–4]. The integration not only can compensate for the disadvantage of a single function in situ and provides an attractive strategy for tumor treatment with simplified procedures [5, 6], but also can improve the tumor identification efficiency, real-time tracking the in vivo distribution of nanoparticles, and continuous monitoring of antitumor therapeutic effect due to the excellent imaging capability [7–9]. Among the currently available imaging modalities, Raman imaging has exhibited excellent advantages in tumor-bearing mice models due to its high sensitivity and strong specificity [10–12]. The surface-enhanced Raman scattering (SERS) enhancement factor caused by the localized surface plasmon resonance is usually 10^7^—10^14^, even allowing the detection of single-molecule [13, 14]. Gold nanoparticles are commonly used for Raman imaging due to its strong localized surface plasmon resonance [15, 16]. Moreover, gold nanoparticles also show high optical absorbance in the near-infrared (NIR) region (700–1000 nm), which enabling them to have the ability of photoacoustic (PA) imaging on the one hand, and on the other hand can increase the tumor local temperature and kill cancer cells under the laser irradiation, which allowing them to serve as an ideal PA imaging and photothermal therapy (PTT) agent simultaneously [17–20]. Therefore, the gold nanoparticle-based theranostic agent could be considered as an optimal in situ tumor detection and monitor of therapeutic response using Raman/PA bimodal imaging, and effective antitumor treatment under the NIR laser irradiation.

The delivery and accumulation efficiency of nanoparticle-based theranostic agents into tumors are the main influencing factors for the tumor diagnosis and treatment [21]. However, the efficiency in nanoparticle delivery and uptake is principally caused by the complex interaction between transport barriers forced by the tumor microenvironment and the existence of tumor-associated macrophages [22–25]. Typically, the delivery and selective accumulation of nanoparticles in solid tumors are largely attributed to the “passive targeting” by enhanced permeability and retention (EPR) effects [26, 27]. Unfortunately, in most cases, more than 95% of NPs are failed to arrive at the tumor site, owing to the hinder of tumor microenvironmental barriers, such as the dense interstitial structure of tumor, poor vascular permeability, and lacking vessels in tumor [28–30]. One conventional strategy for overcoming this matter is attaching active targeting ligands or antibodies on the surface of NPs, for improving the targeting of NPs to tumor cells [31, 32]. Nevertheless, some researchers revealed that the percentage of active-targeted injection dose (% ID) has increased by only about 50% compared to passively accumulated NPs, and the improvement for clinical trials is generally ambiguous [29, 30, 33]. The above issues underscore that tumor microenvironment including vascular perfusion, permeability, and density are major influencing factors for efficient delivery and accumulation of NPs in tumors [34, 35]. Therefore, more efforts need to do for improving the delivery and accumulation efficiency of nanoparticle-based theranostic agents and further improving the antitumor effect. Recent studies have established that X-ray irradiation in the local tumor showed a strong influence on intratumoral delivery and bulk accumulation of the theranostic agents especially for the vascular permeability and density, which is a promising strategy to improve the accumulation of NPs in tumor site by changing the interstitial fluid pressure, vascular permeability and density [29, 36]. Stapleton et al. reported that single dose of radiation can substantially improve the tumor uptake and distribution of nanoparticles, and them improve the therapeutic effect [37]. Kim and coauthors found that tumor irradiation could enriched tumor associated macrophages in the tumor interior, then, can increase higher tumor drug delivery and drug penetration [38]. Yang group reported that the tumor accumulation and retention much of human serum albumin nanoparticle can significantly improve by X-rays exposure of the tumor [39]. Therefore, the irradiation of tumor tissue can assist the uptake and retention of nanoformulation owing to the local change of interstitial fluid pressure, vascular permeability and density.

**Scheme 1:**
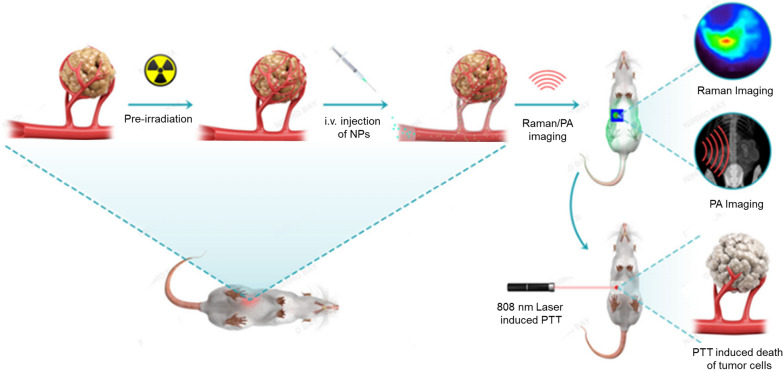
Scheme of Pre-irradiation mediated tumor vascular proliferation for enhanced nanoparticles delivery, dual-modal imaging and the photothermal therapy (PTT) of the tumor

Herein, we report a biocompatible theranostic nanoparticle termed as HAuNP@DTTC via the preparation of hollow gold NPs coupled with Raman reporter, 3.3-diethylthiatricarbocyanine iodide (DTTC), which integrate the Raman/PA imaging and PTT on a single nanoplatform. What is particularly exciting is that the efficiency of nanoparticles delivery and accumulation at the tumor site could be increased significantly, and then the imaging and therapy effects could also be enhanced under the adjuvant of low-dose X-ray pre-irradiation (Pre-IR) (Scheme 1). The in vivo results have further demonstrated that with adjuvant of low-dose (3 Gy) X-ray irradiation, not only the tumor Raman/PA imaging was significantly enhanced, but the tumor growth inhibition (TGI) of HAuNP@DTTC-induced PTT was also increased from 60% for PTT only to 97% by using a 4T1 breast cancer-bearing mice model. We also investigated the mechanism of the bulk accumulation of HAuNP@DTTC by PA and Raman imaging technique and immunohistological staining, which showed that the X-ray-induced vascular proliferation is the main factor for the high delivery and accumulation efficiency of HAuNP@DTTC. These results revealed a potential translational strategy by combining HAuNP@DTTC with X-ray irradiation for cancer diagnosis and image-guided PTT. In general, we anticipate that the X-ray irradiation-induced improvement of the efficiency of nanoparticles delivery and accumulation would put an avenue to inspire more researchers to design more nano-agents for tumor theranostics.

## Materials and methods

### Synthesis of HAuNP@DTTC

The HAuNP@DTTC were synthesized by two main steps: Firstly, the Ag nanoparticles were prepared by co-reducing AgNO_3_ (1 wt%, 0.25 mL) with ascorbic acid (AA, 10 mM, 0.7 mL) and sodium citrate (1 wt%, 1 mL) in boiling water (47.5 mL), and then the suitable volume of HAuCl_4_ solution (100 mM) was added at room temperature to form hollow Au nanoparticles (HAuNP) with appreciate LSPR absorption peak around 808 nm. Secondly, the Raman tag (3,3′-diethylthiatricarbocyanine iodide, DTTC) solution was incubated with prepared hollow Au nanoparticles and followed by bovine serum albumin (BSA) modification for one night and the suspension was centrifuged to remove free DTTC and BSA. The HAuNP@DTTC were obtained after re-suspending in deionized water for further use.

### Pre-irradiation treatment

For all the irradiation treatment, a 160 kV PXi X-ray irradiation apparatus (X-RAD160, PXi, USA) was used. The average dose of the X-ray was calculated by the dosimetric studies using standard dosimeter techniques showing that in the center of the aperture to be 68.5 ± 3% (mean ± SD) of that delivered with a fully open field. Before irradiation, mice were anesthetized via 2% ~ 3% isoflurane, immobilized on the platform with surgical tape, and irradiated individually under low-dose x-ray (3 Gy per mouse). In order to protect the normal tissue around the tumor in mice, a small lead protective box made by ourselves was used during the irradiation.

### Photoacoustic (PA) imaging

For the acquiring and recording of PA images, a Vevo Photoacoustics Imaging System (Visual Sonics, Toronto, Canada) was used. The in vitro PA signal spectra of the HAuNP@DTTC were obtained (690 to 950 nm with a 5 nm interval) for subsequent experiments. The in vivo PA images of the tumor regions were performed with 4T1 tumor-bearing mice at different time points. Before imaging the mice were intravenously injected with HAuNP@DTTC (concentration = 100 μg/mL) dispersed with 150 μL PBS, then the mice were anesthetized with isoflurane.

### Surface-Enhanced Raman Scattering (SERS) imaging

All the SERS images (including the NPs and tumor-bearing mice) were performed by a Renishaw Invia Reflex Raman microscopy system, equipped with a 785 nm laser beam directed to the sample through an objective lens (20 × or 50 × long working distance NA = 0.75) to focus the laser onto the sample then collecting the SERS signals of HAuNP@DTTC from the samples through a CCD camera. The Raman system was calibrated by reference to a silicon wafer at the vibrational band of 520 cm^−1^ before all measurements. SERS mapping was performed in a rectangular area that covered the entire tumor region and the laser point was moved step by step (50% laser, 0.5 s integration time per step). After all the in vivo SERS images at each time point were collected, the mice were sacrificed, the tumor region excised and fixed with 4% paraformaldehyde (PFA) for the other experiments.

### Photothermal imaging and PTT

The tumor growth delay study was carried in orthotopic 4T1 tumor-bearing mice. When the tumor volume reached 75 mm3, mice were randomly allocated into the 5 groups: (1) control (150 μL PBS), (2) Pre-IR (3 Gy X-ray only), (3) Pre-IR + NPs (3 Gy X-ray + 150 μL PBS), (4) PTT (150 μL NPs + 808 nm laser), and (5) Pre-IR + PTT (3 Gy X-ray + 150 μL NPs + 808 nm laser) groups (n = 5 per group). The mice in the PTT group were treated with 808 nm laser (2.0 W cm^−2^, 3 min) 24 h post intravenous injection of NPs. And for the Pre-IR + PTT group, the mice were exposed to the X-ray irradiation (3 Gy per mouse) 3 days before NPs injection, after then the mice were treated under 808 nm laser for PTT. The temperature changes of the tumor region were monitored by using a thermal camera (FLIRA300, FLIR System, Wilsonville, Oregon, USA). The tumor size was measured with a digital caliper every 2 to 3 days after various treatments, and the tumor volumes were calculated as volume (mm3) = length × width^2^ × 0.5. For photothermal effect studies, HAuNP@DTTC dispersions were treated by using 808 nm laser at different energy densities (0.2, 0.5, 0.8, 1.0, and 1.5 W cm^−2^ for 5 min), during which time the temperature of NP dispersions was monitored continuously.

## Results and discussion

### Synthesis and characterization of HAuNP@DTTC

Hollow Au nanoparticle coupled DTTC (HAuNP@DTTC) was synthesized through two procedures including the synthesis of HAuNP and the assembly of HAuNP@DTTC. The HAuNP were produced via a one-step procedure, in which the Ag nanoparticles was selected as the sacrificial template to reduce the HAuCl_4_. And then, DTTC, a common infrared dye molecule, could be tagged onto the surface of HAuNP as the Raman reporter [40, 41], assisted with the BSA molecule as a stabilizer [42, 43] to achieve the HAuNP@DTTC eventually.

**Fig. 1:**
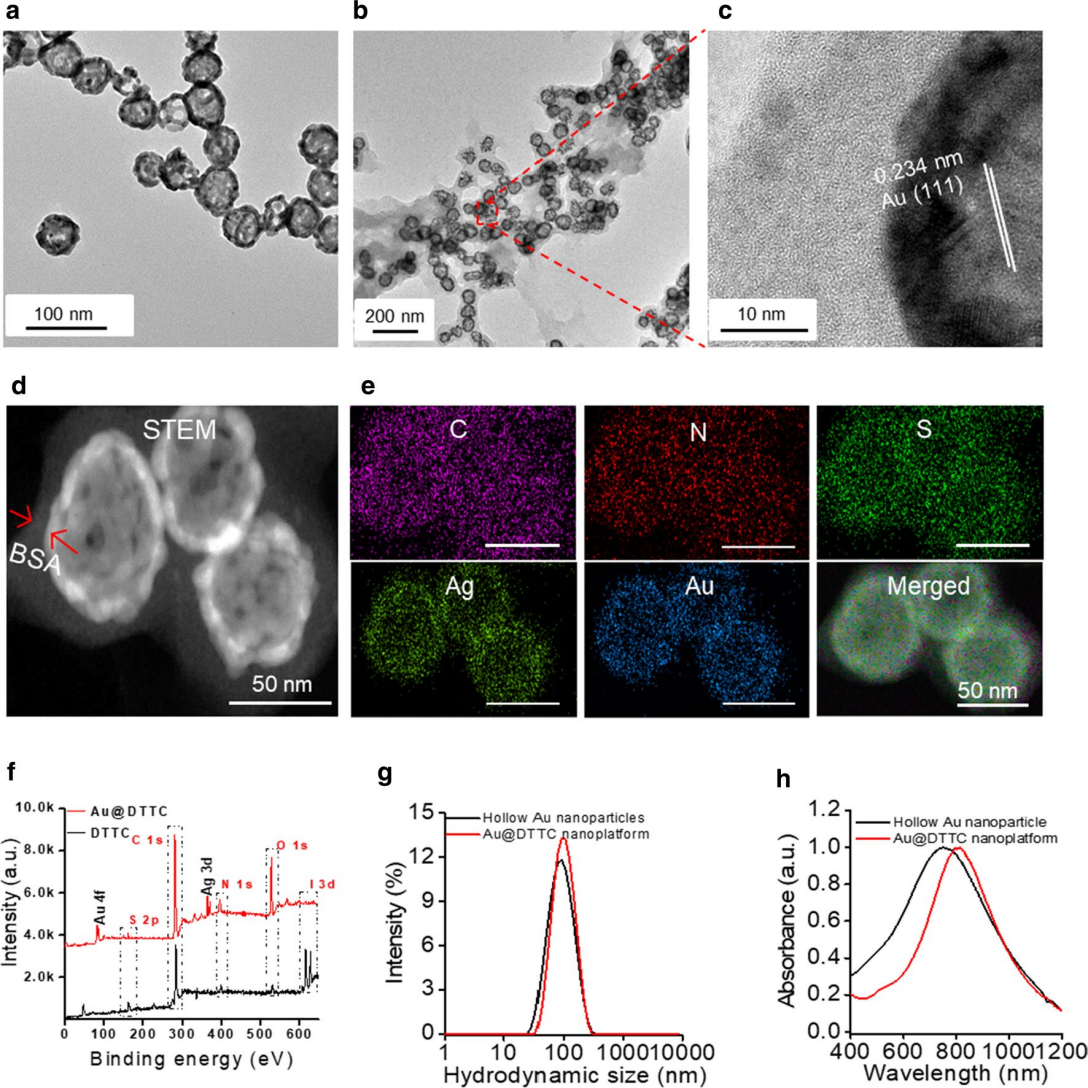
In vitro characterization of prepared Hollow Au nanoparticle-coupled DTTC (HAuNP@DTTC). **a** TEM images of as-synthesized HAuNP. **b** TEM images and **c** the high magnification of HAuNP@DTTC, the white lines denote the lattice spacing of Au. **d**, **e** Localized STEM image of HAuNP@DTTC and the corresponding EDS mapping images. **f** XPS analysis of HAuNP@DTTC (red line) and DTTC (black line). **g** DLS curves of HAuNP and HAuNP@DTTC. **h** UV–vis absorption spectra of HAuNP and HAuNP@DTTC

The transmission electron microscopy (TEM) image in Fig. 1a showed that the morphology of the synthetic Au nanoparticles is hollow sphere (named HAuNP). After the modification of DTTC and BSA, a translucent thin shell was coating on the surface of HAuNP (Fig. 1b, c), and the lattice space (0.234 nm) of nanoparticles is consistent with Au (111) in the high magnification TEM image (Fig. 1c). This result demonstrated that the coating of DTTC and BSA did not affect the structure of HAuNP. The scanning transmission electron microscopy (STEM) (Fig. 1d) and elemental mapping of N, S elements (Fig. 1e) confirmed that the HAuNP was surrounded with BSA polymer shell. The X-ray photoelectron spectroscopy (XPS) spectra of the synthesized HAuNP@DTTC and DTTC molecules were recorded (Fig. 1f), the characteristic peaks located at ~ 161, 282, and 615 eV are belonging to S, C, O, and I elements, further proved that DTTC and BSA were successful assembled with HAuNP. The DLS result (Fig. 1g) showed that the average hydrodynamic diameters increased from ~ 70 nm for HAuNP to ~ 85 nm after the surface modification with DTTC and BSA, while the zeta potential (Figure S1c) of HAuNP@DTTC had a slightly increase. No obvious size change was observed in different physiological solutions (H_2_O, PBS, FBS, and DMEM + 10% FBS) for 7 days (Additional file 1: Fig. S2).

HAuNP@DTTC still maintain the LSPR property of HAuNP in the near-infrared (NIR) range (Fig. 1h), which had a broad absorption in the range of 500—1100 nm, and with a maximum absorption peak at 813 nm. The strong optic absorbance of HAuNP@DTTC around 808 nm, given them the excellent ability to realize the NPs mediated photothermal therapy (PTT) and photoacoustic (PA) imaging under the irradiation of NIR laser [44–47].

### In vitro function evaluation of prepared HAuNP@DTTC

**Fig. 2:**
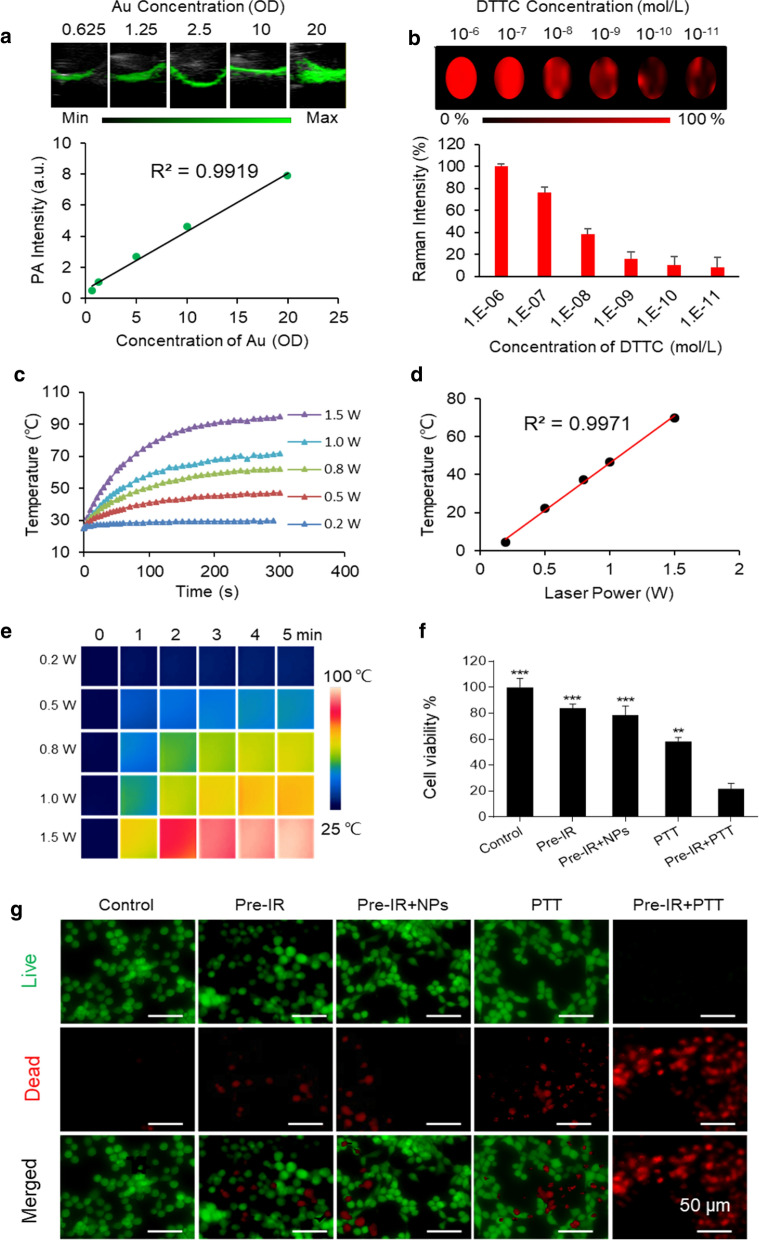
In vitro function evaluation of prepared HAuNP@DTTC. **a** PA images of HAuNP@DTTC with a different concentration in PBS (up), and the relationship between the concentration of HAuNP@DTTC and PA intensity (down). **b** Raman images of HAuNP@DTTC with different DTTC concentration (from 10^–6^ to 10^–11^ μmol L^−1^) of Raman reporter (up), and corresponding Raman intensities (down). **c** Photothermal profiles of HAuNP@DTTC in PBS (100 μg mL^−1^) under different power density irradiation (808 nm laser). **d** Infrared thermal photographs of HAuNP@DTTC (100 μg mL^−1^) under different power density irradiation (808 nm laser, 5 min). **e** Relationship between max temperature and power density. **f** Cell viabilities of 4T1 cells after different treatments. **g** The viabilities of 4T1 cells in the different treatment groups by Calcein-AM/PI staining

To explore the application potential of our synthesized HAuNP@DTTC, we investigated the in vitro imaging and therapy functions. For the evaluation of PA imaging, a series of HAuNP@DTTC dispersions at different concentrations (0.625 to 20 OD) were prepared and scanned (Additional file 1: Fig. S2). Representative PA images and the corresponding linear relationship between PA intensity and HAuNP@DTTC concentrations are shown in Fig. 2a, indicating an excellent concentration-dependent PA intensity enhancement. Then we investigated the Raman imaging of HAuNP coupled with different DTTC concentrations (from 10^–6^ to 10^–11^ M). The red Raman images (up, Fig. 2b), the corresponding Raman intensities bar chart (down, Fig. 2b) and the Raman spectra in Additional file 1: Fig. S3, showed that although the Raman signal intensities reduced with decreasing of DTTC concentration, but the Raman signals could still be detected when the concentration drops to 10^–11^ M. Also, the Raman image was clear even if the DTTC concentration decreased to 10^–11^ M, revealing the highly sensitivity Raman imaging characteristics of HAuNP@DTTC. These findings demonstrate that HAuNP@DTTC can not only be used as a PA imaging agent but also has the potential applications as a Raman-active imaging probe.

Furthermore, we studied the impact of power density on the HAuNP@DTTC-induced hyperthermia. HAuNP@DTTC dispersion in water (5 OD, ~ 100 μg mL^−1^) was irradiated under 808 nm laser with different power density (0.2, 0.5, 0.8, 1.0, and 1.5 W cm^−2^) for 5 min. The temperature changes curves (Fig. 2c) and the color-coded images (Fig. 2d) show that HAuNP@DTTC experienced obviously temperature increases within 2 min under different power density irradiation. The temperature changes (Fig. 2e) of HAuNP@DTTC dispersion irradiated with this series of power density, suggesting that a minimal 0.8 W cm^−2^ could heat the dispersion above 42 °C after 2 min of laser irradiation, which is sufficient enough for irreversible apoptosis of tumor cells owing to the hyperthermia. Considering the disparity of in vitro and in vivo, we chose 1.0 W cm^−2^ for the following research to ensure the in vivo therapy effect.

The cell viability and PTT effect on the tumor cells (4T1) of HAuNP@DTTC were assessed by MTT assays. Cell viabilities were above 85% for 4T1 tumor cells even at high concentrations after co-incubation for 24 h, demonstrating low toxicity of HAuNP@DTTC without laser irradiation (Additional file 1: Fig. S4). With the adding of laser irradiation (PTT, 808 nm, 1.0 W cm^−2^) and/ or low-dose X-ray pre-irradiation (Pre-IR, 3 Gy), however, HAuNP@DTTC displayed favorable toxicity on 4T1 cells (Fig. 2f). The combination of HAuNP@DTTC and 808 nm laser killed 48 ± 2.5% of 4T1 cells compared to HAuNP@DTTC alone; the Pre-IR alone and combination of Pre-IR and HAuNP@DTTC killed 15 ± 1.8% and 21 ± 3.9% of 4T1 cells, respectively, which means that the low-dose X-ray did not induce obvious cytotoxicity; while over 92 ± 1.9% of 4T1 cells died after the Pre-IR + HAuNP@DTTC + laser (Pre-IR + PTT) treatment. Calcein-AM/PI co-staining (Fig. 2g) of 4T1 cells after the above treatments showed a similar result, more than 94% of 4T1 cells were PI-positive (red) after Pre-IR + PTT.

### Bulk accumulation of HAuNP@DTTC after the pre-irradiation of low-dose X-Ray

**Fig. 3:**
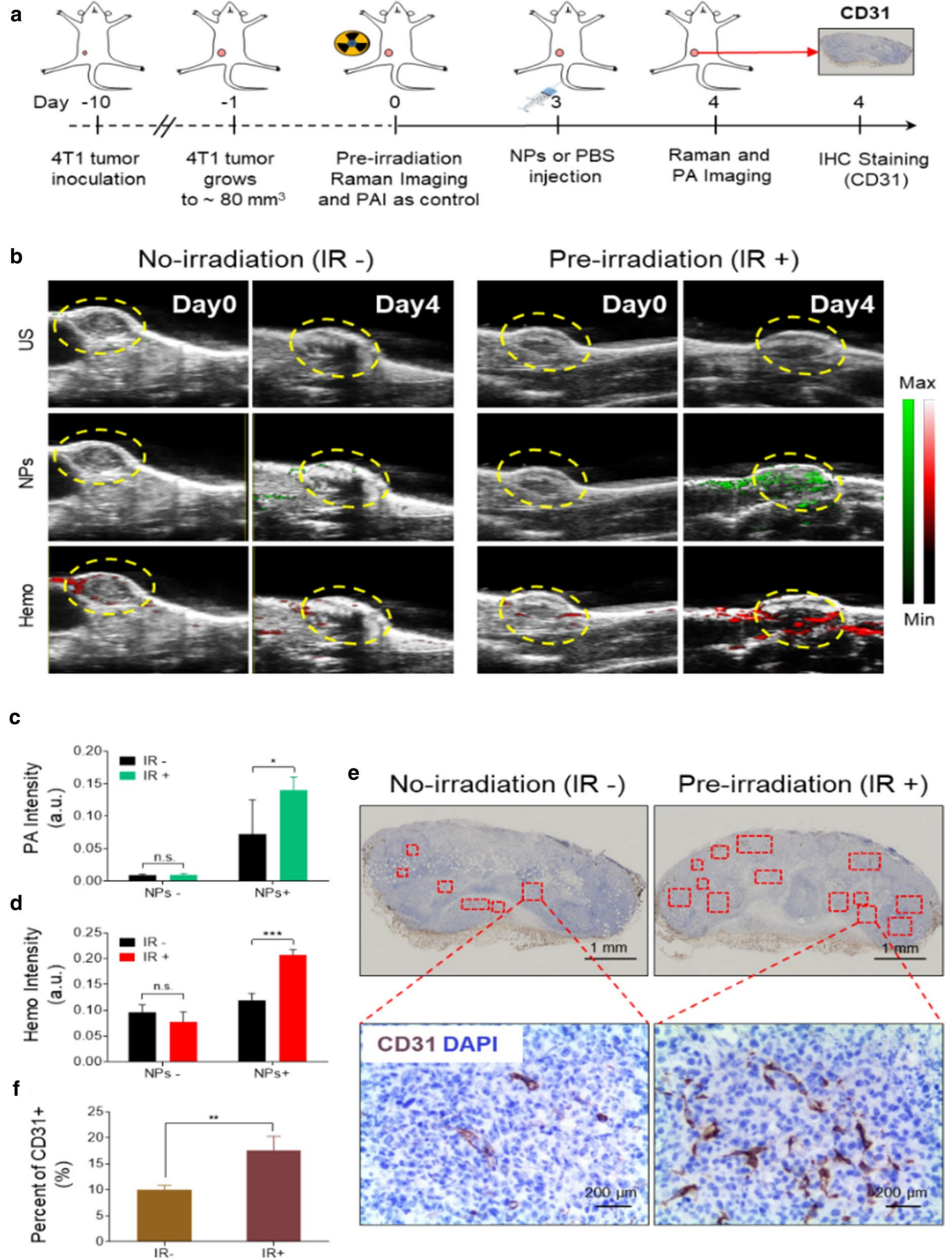
Pre-irradiation induced the vascular proliferation and improved the bulk accumulation of HAuNP@DTTC in tumors. **a** Schematic illustration of Pre-irradiation enhanced accumulation of HAuNP@DTTC in tumors for the tumor diagnosis under Raman/PA dual-model imaging. **b** Representative ultrasound (US) and photoacoustic (PA) images of mice bearing 4T1 tumors with (right panel) and without (left panel) irradiation, day 0 and day 4 represents the images before and after the injection of HAuNP@DTTC, and the third line of (B) showed the oxyhemoglobin (Hemo) saturation levels in the tumors, yellow dash circles indicated the tumor sites. **c**, **d** Quantification of PA intensities from HAuNP@DTTC and Hemo saturation levels in the tumors based on the images shown in (**b**), the pre-irradiation increased the HAuNP@DTTC accumulation and the Hemo saturation levels were augmented. **e** Representative images of CD31 and DAPI staining of tumor cross-sections with and without pre-irradiation after 4 days, the red dash boxes denote the CD31 positive (CD31 +) regions. **f** Quantification of the percent of CD31 + regions in the tumor sections based on the images shown in (**e**), there was a significant increase in CD31 staining in the mice treated with irradiation (3 Gy). Statistically significance was calculated via one-way ANOVA with a Tukey post-hoc test, ^*^p < 0.05; ^**^p < 0.01; ^***^p < 0.005

The efficiency of delivering nanotherapeutics to the tumor site plays a decisive role in increasing the anti-tumor efficacy. Recent studies pointed out that X-ray irradiation can improve the vascular permeability, which is a key driving factor that can affect the delivery of nanotherapeutics positively and negatively [29, 36]. As the adding of low-dose X-ray irradiation (3 Gy) have exhibited the good cell killing effect in the above section, we then studied the influence of X-ray pre-irradiation (Pre-IR) on the orthotopic 4T1 breast cancer model (the treatment schedule is shown in Fig. 3a). Representative ultrasound and PA imaging are performed before HAuNP@DTTC injection without (IR-, left, Fig. 3b) and with (IR + , right, Fig. 3b) X-ray treatment at day 0, no emerging of PA signals and the hemoglobin saturation (HbO_2_) levels of the tumor regions are relatively low. The HAuNP@DTTC dispersion in PBS (100 μg/mL, 200 μL) was injected 3 days after different treatment (no X-ray treatment for IR- and 3 Gy X-ray treatment for IR +). Then the PA imaging was performed again 24 h post-injection. As demonstrated in Fig. 3b, the PA intensity and the HbO_2_ levels of the IR- group only had a slight increase; while the PA image of the IR + group was clearer on day 4, which could diagnosis the tumor more accurately. The HbO_2_ level also increased notably, demonstrated the increase of oxygenated blood, which due to the increased blood flow at tumor site. In addition, the changes of PA images that due to the improved accumulation of HAuNP@DTTC in the tumor site, providing a strong proof that assisting of low-dose X-ray irradiation in the tumor site is an enhancement factor for NPs delivery. A consistent trend of the intensities of PA and HbO_2_ was obtained from the quantitative analysis of the PA images **(**Fig. 3c) and HbO_2_ images (Fig. 3d). For example, the PA intensity of the tumor has a significant increase with the assisting treatment of low-dose X-ray irradiation, where the *p < 0.05. These results demonstrated preprocessing with low-dose X-ray could increase the accumulation of NPs in to tumor site, which will be very beneficial for improving the effect of tumor diagnosis and treatment.

It is known that the blood vessels in the tumor play a major role in the delivery and accumulation of drugs and nanotheranostic agents [30], and the X-ray irradiation can affect the vascular conditions. Therefore, the main underlying biological mechanism of X-ray irradiation-induced bulk accumulation of HAuNP@DTTC includes the vascular burst, proliferation, or both of them were investigated [29, 36, 48]. Given the increase of HbO_2_ level in the tumor after X-ray irradiation above, we speculate that the improved accumulation is mainly caused by the vascular proliferation. To further test this hypothesis, the mice were sacrificed at 24 h post-HAuNP@DTTC administration, and the tumor tissue was harvested of each group (IR-: no X-ray, IR + : 3 Gy X-ray) for immunofluorescence staining and analysis. Endothelial-specific CD31 was chosen to quantify the vascular density, which is a common indicator for assessing tumor angiogenesis [49, 50], and DAPI for nuclear staining. We found considerable changes in the expression of CD31 following 3 Gy X-ray irradiation, compared to control (Fig. 3e). And the quantification analysis of CD31 staining (Fig. 3f) also verified this result. Therefore, it can be determined that the improved bulk accumulation of HAuNP@DTTC is mainly caused by vascular proliferation. Besides, we can conclude that low-dose X-ray irradiation is an important influencing factor for changing of vascular density in tumor, and provide a promising enhancement method for nanotheranostic agents delivery.

### Improved Raman imaging due to the augment of HAuNP@DTTC

**Fig. 4:**
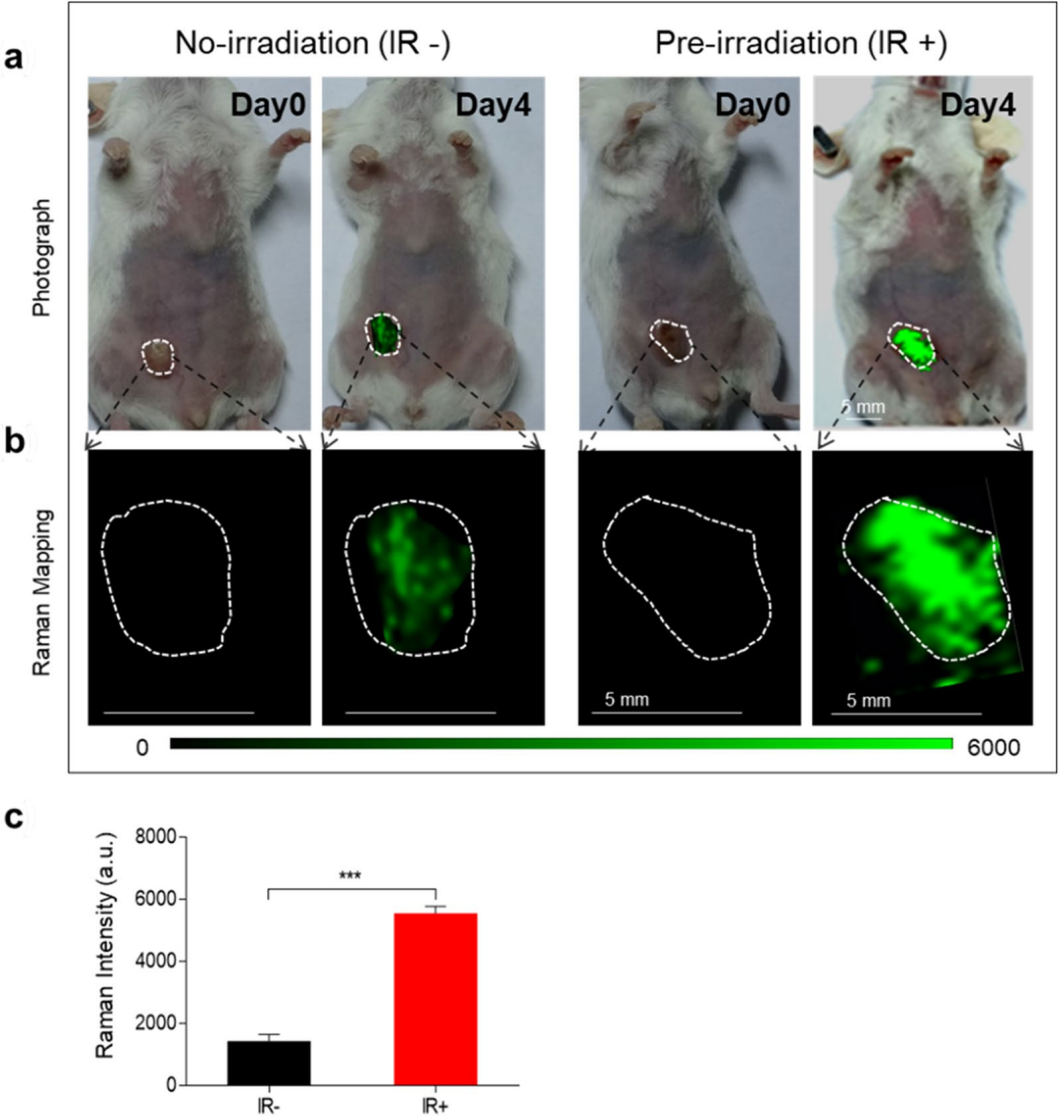
Increasing HAuNP@DTTC accumulation improved the Raman imaging effect of tumors. **a** Photograph of the mice bearing 4T1 tumor with (right) and without (left) irradiation treatment, day 0: before HAuNP@DTTC injection, day 4: 24 h post HAuNP@DTTC injection (100 μg/mL, 200 μL). **b** The Raman mapping images of the tumors in the two different treatment groups, white dash circles denoted the tumor regions. **c** Quantification of the Raman intensities on day 4 in two different groups. Statistically significance was calculated via student T-test, ^***^p < 0.005

Raman imaging is a novel diagnostic approach that uses noble metal nanoparticles for SERS, resulting in highly sensitivity (fM) and specificity (finger-like spectrum) imaging [51, 52]. The in vitro results (Fig. 2b) have verified that HAuNP@DTTC exhibited an excellent imaging capability for Raman imaging. Furthermore, we studied the in vivo Raman imaging capability after injecting HAuNP@DTTC (100 μg/mL, 200 μL) into 4T1-bearing mice via the tail vein. The photograph in Fig. 4a showed the tumor site, and the acquired Raman images in Fig. 4b at day 0 and 4 in the tumor region exhibited a significant increase with the adding of X-ray irradiation. That is, the tumor can be outlined through Raman imaging and which will be more clearly following the adding of X-ray irradiation. Quantification analysis (Fig. 4c) indicated that around 3 ~ fourfold higher of the Raman intensity in X-ray treated 4T1-bearing mice than no X-ray adding group. Then we harvested the tumor tissues and prepared for TEM characterization. The Raman images and TEM images (Additional file 1: Fig. S5), provided another clear evidence that X-ray can improve the delivery and accumulation of HAuNP@DTTC to the tumor, and provided a new promising imaging technology for tumor diagnose.

### In vivo imaging-guided photothermal therapy (PTT) of HAuNP@DTTC

**Fig. 5:**
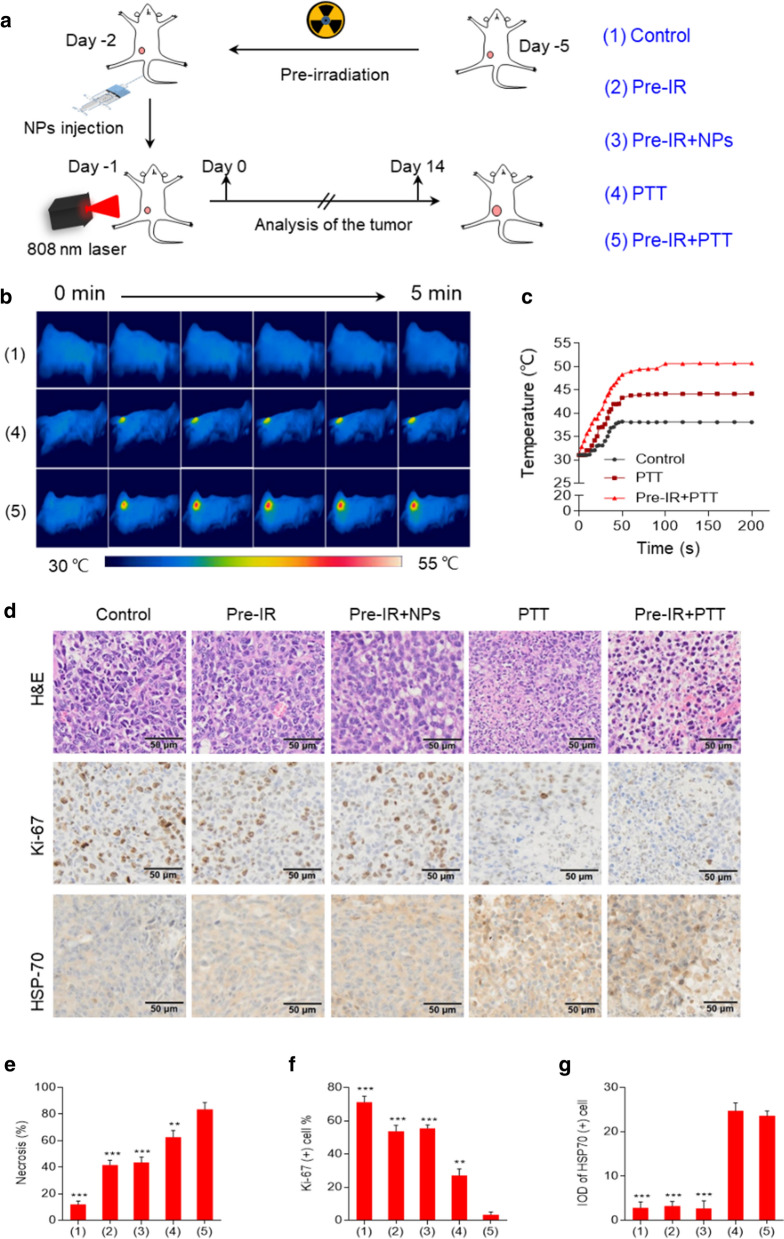
Short-term in vivo photothermal therapy (PTT) treatment after HAuNP@DTTC injection. **a** Schematic illustration of the in vivo anti-tumor experiments design. **b** Representative infrared thermal images of 4T1-bearing mice in control, PTT, and Pre-IR + PTT groups (24 h after *i.v.* injection; 808 nm laser under 1.0 W cm-2 for 5 min; n = 3, per group). **c** Temperature elevation at the tumor site after near-infrared laser treatment. **d** Representative H&E, Ki-67, and HSP-70 staining of tumors at 48 h after various treatments, Scale bars: 50 μm. **e**–**g** Percentages of necrosis, Ki-67 positive cells and HSP-70 positive at 48 h after various treatments. ^*^p < 0.05, ^**^p < 0.01, ^***^p < 0.01

Imaging-guided photothermal therapy will provide more advising for tumor treatment[53–57]. Due to the excellently improved tumor accumulation of HAuNP@DTTC with X-ray irradiation, we rationally evaluated the anti-tumor efficacy of HAuNP@DTTC mediated PTT with the assist of X-ray in vivo in 4T1 tumor-bearing mice, which were randomly assigned to five treatment groups (n = 3): (1) control, (2) Pre-IR, (3) Pre-IR + HAuNP@DTTC (Pre-IR + NPs), (4) PTT, (5) Pre-IR + PTT, the treatment schedule for BALB/c mice is shown in Fig. 5a. Thermal images of the tumor region captured by using an infrared thermal camera (Fig. 5b, c) revealed that the surface temperature of the tumors treated with PTT after X-ray irradiation reached 52 °C (Pre-IR + PTT, 3 Gy, 808 nm, 1.0 W cm^−2^, 5 min), and the surface temperature of tumors treated with PTT only maintained at 43 oC. In contrast, the temperature of the tumor surface was only increased to around 38 °C for PBS-injected mice after the same exposure time. The tumor tissues were collected 48 h-post all the different treatments and fixed with 4% PFA for studying the variations of cell apoptosis, cell proliferation and cell thermal response in tumors by using immunohistochemical staining (Fig. 5d-g). From the images of hematoxylin and eosin (H&E) and Ki-67 stained tumor slices (Fig. 5d, e), whereas about 62% of the tumor cells in the mice of the PTT only group showed necrotic status, and the necrotic tumor cells increased to ~ 84% after the adding of X-ray. The tumor cells in control, Pre-IR, and Pre-IR + NPs were only partially destroyed. Similarly, the proliferation of tumor cells (Ki-67 positive) in the case of the Pre-IR + PTT group was largely inhibited as a result of the favorable delivery and accumulation of HAuNP@DTTC in tumors, compared with other groups. Moreover, about 22% ~ 26% of HSP-70 positive cells (Fig. 5g) were observed in the 808 nm laser performed groups.

**Fig. 6:**
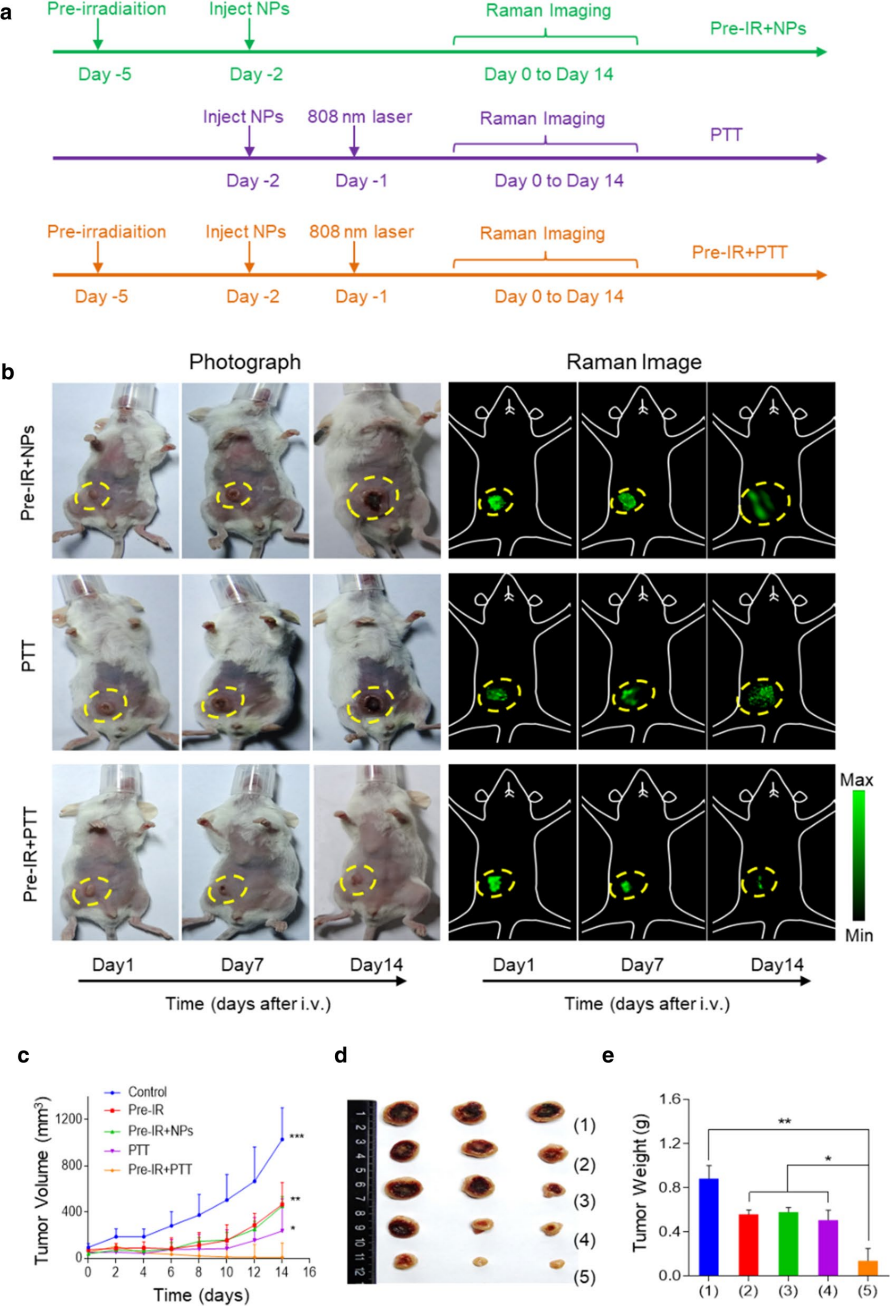
Monitoring the anti-tumor effect of HAuNP@DTTC in combination with Pre-IR and PTT by Raman imaging technique in 4T1-bearing mice. **a** The scheme of the design for the Pre-IR and PTT combination therapy. **b** Monitoring the tumor growth situation by photographs (left) and Raman images (right) of the 4T1-bearing mice in each group until 14 days after various treatment, the yellow dash circles denote the tumor sites. **c** Tumor growth curves of mice bearing 4T1 tumors followed by different treatments. **d** Representative photograph of dissected tumors. **e** The weights of dissected tumors from different groups. Statistically significance was calculated via one-way ANOVA with a Tukey post-hoc test, ^*^p < 0.05; ^**^p < 0.01; ^***^p < 0.005

Then we carried out the in vivo tumor growth delay study on the 4T1-bearing mice to evaluate the therapeutic efficacy of HAuNP@DTTC combined with 808 nm laser irradiation after the adjuvant of low-dose X-ray, and monitored the tumor growth through Raman imaging technique (the treatment schedule is shown in Fig. 6a). As seen from the Raman images in Fig. 6b, the Raman signal intensity of the tumor site (circled by the yellow line) increases 24 h post-injection then decreases with the growth or diminish of the tumor in the following days, while the area of the Raman images is consistent with the tumor volume (Fig. 6c), revealing that Raman imaging technique can be used as a tool for monitoring the anti-tumor efficacy. For instance, tumors were effectively eliminated post-treatment in the Pre-IR + PTT group, the corresponding Raman signals in Fig. 6b is also hard to detect, demonstrating improved anti-tumor efficacy by a combination of X-ray irradiation, injection of HAuNP@DTTC and 808 nm laser. The representative tumor images (Fig. 6d, Additional file 1: Fig. S6) and tumor weight (Fig. 6e) after 14-days initiation treatment further revealed that the Pre-IR + PTT-treated group showed the optimal tumor killing effect, affirming the adjuvant of X-ray strongly enhanced the PTT in tumors. With the adding of X-ray, tumor growth inhibition (TGI) showed the same trend and increased from 60% for PTT only to 97% for Pre-IR + PTT (Additional file 1: Table S1).

### Pulmonary and hepatic metastasis evaluation

**Fig. 7:**
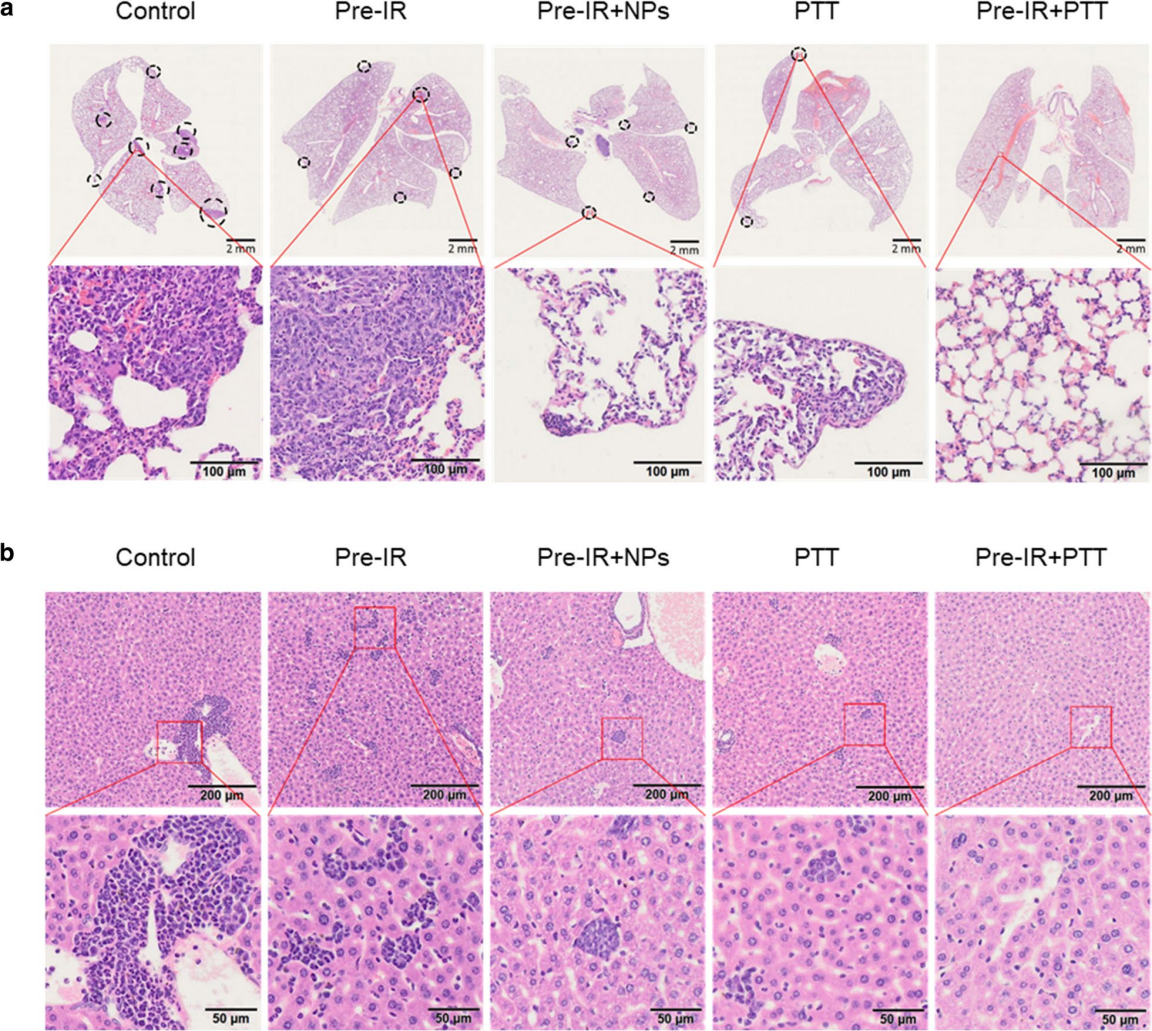
Anti-metastasis of HAuNP@DTTC in the combination of Pre-IR and PTT in orthotopic 4T1-bearing mice. Representative hematoxylin–eosin (H&E) of the lung (**a**) and liver (**b**) of each treatment group

Lethal tumor metastasis often occurred at the late stage in breast cancer patients, mainly due to the dissemination of cancer cells to distant sites such as the lung and liver [58–60]. In our work, a large number of lung metastatic lesions were shown in mice in control, Pre-IR, and Pre-IR + NPs groups on day 14 (Fig. 7a). Fewer metastatic lesions were found in the PTT group, demonstrating the treatment could inhibit the tumor metastasis, but not enough for preventing all the tumor invasion. In contrast, no visible lung metastatic lesion was observed in the combination of X-ray and PTT treatment. The H&E staining of the liver for the evaluation of hepatic metastatic received a similar result, the X-ray adjuvant treatment combining with PTT significantly reduced the micro-hepatic metastases (Fig. 7b). Taken together, these outcomes further confirmed that X-ray-assisted PTT could effectively inhibit distant metastasis of 4T1-bearing mice.

### Preliminary toxicity study

**Fig. 8:**
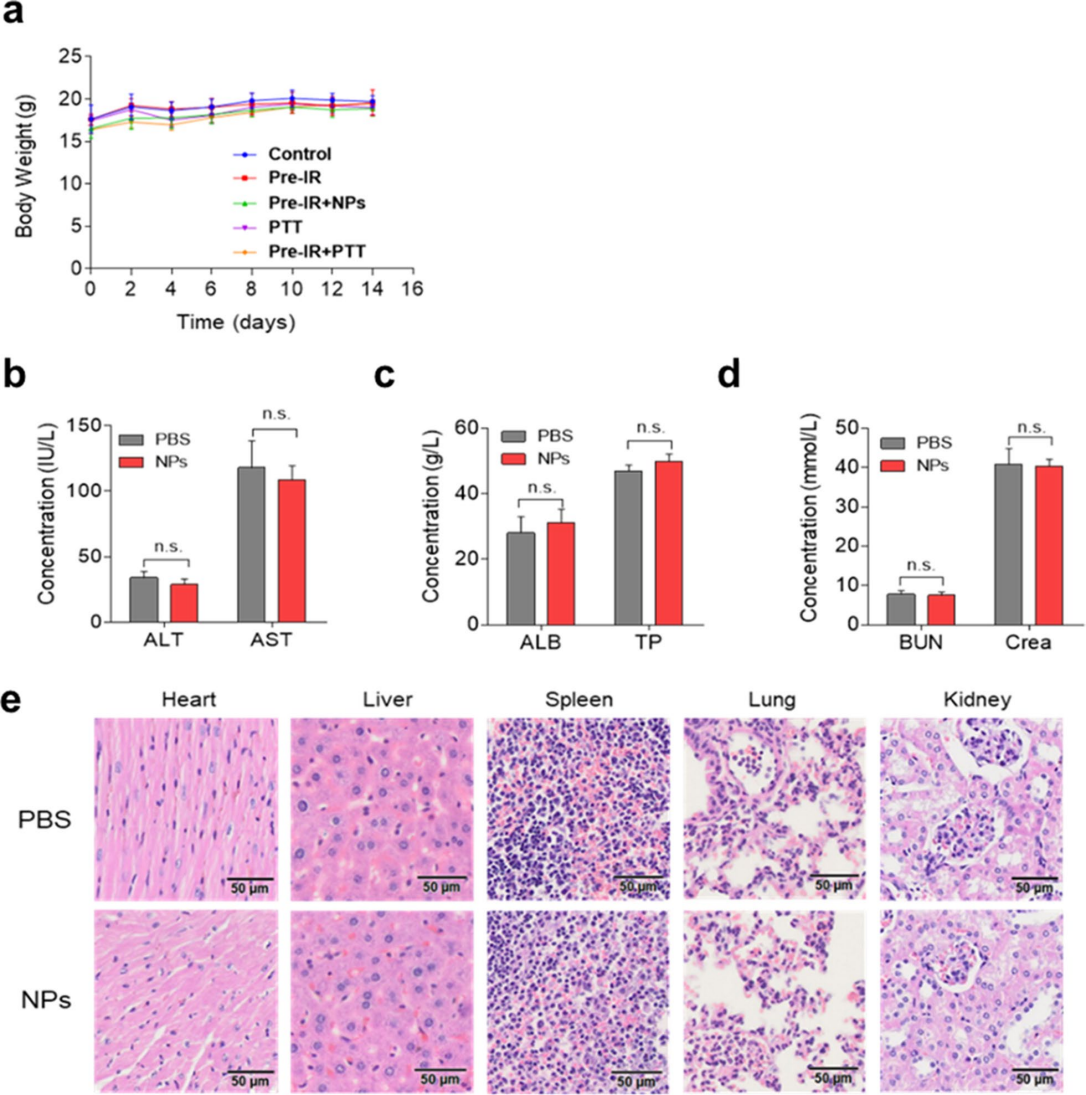
Preliminary toxicity study of HAuNP@DTTC. **a** Body weight of mice in each treatment group (n = 5). **b**–**d** Blood biochemistry tests 30 days after the PBS and HAuNP@DTTC injections via the tail vein. **e** Histology analysis of major organs after PBS and HAuNP@DTTC injections. No obvious toxicity of the major organs was found after the treatments

For the preliminary toxicity analysis, we found no body weight changes during the treatment (Fig. 8a). In addition, the administration of HAuNP@DTTC did not influence the alanine aminotransferase (ALT), aspartate transaminase (AST), albumin (ALB), total protein (TP), blood urea nitrogen (BUN), and creatinine (CREA) as demonstrated in Fig. 8 b-d. Histological examination of major organs (Fig. 8e) (heart, liver, spleen, lung, and kidney) demonstrated no significant organ damages or inflammatory lesions. Moreover, we performed the hemolysis test (Additional file 1: Fig. S7), which demonstrated the low toxicity of HAuNP@DTTC NPs to the human body. Taken together, these results demonstrated that with the assistance of X-ray, the accumulation of the HAuNP@DTTC in tumor was improved significantly, and the HAuNP@DTTC’s biosafety in vivo proved that the HAuNP@DTTC can act as an effective anti-tumor nanotheranostic agent with minimal toxicity at the tested dose. However, the toxicity study, for now, is not enough for future clinical translation, and more systematic studies, such as long-term toxicity and immune-compatibility studies, are still necessary.

## Conclusion

In conclusion, we have designed and synthesized the hollow Au nanoparticles-coupled DTTC (HAuNP@DTTC) as a multifunction theranostic agent, which could be used for Raman/PA imaging-guided tumor diagnosis and low-dose (3 Gy) X-ray enhanced PTT. Under the low dose irradiation of X-Ray, the delivery and accumulation efficiency of HAuNP@DTTC in tumor site could be enhanced significantly, and the tumor killing efficiency by PTT has also been greatly improved. The mechanism of it was demonstrated by Raman/PA bimodal imaging technique and immunohistological staining results, that is, the X-ray-induced vascular proliferation is the main factor for promoted delivery and accumulation of HAuNP@DTTC in the tumor. The vascular distribution in the tumor site was changed under the X-ray treatment, revealing the change of the tumor microenvironment. In addition, the HAuNP@DTTC also showed the excellent biosafety in 4T1 cells and mice based on the in vitro and in vivo studies. Therefore, our work provided new insights for enhancing the delivery and accumulation of nanomedicine in tumor site and strong implication potential for future clinical trials of X-ray-adjuvant strategies. While, the X-ray we used for irradiation was kV electrons, which maybe only the most superficial parts of the tumor are permeabilized, and the effects of clinically relevant MV electrons on tumor was not clear and need more comprehensive research in the future.

## Supplementary Information

**Additional file 1**: **Fig. S1**. (a) Hydrodynamic size changes of HAuNP@DTTC nanoparticles in different physiological solutions (H2O, PBS, FBS, DMEM + 10 % FBS) for 7 days; (b) Corresponding photographs of the first and seventh day; (c) Zeta potential of hollow Au, hollow Au with DTTC complex (Hollow Au/DTTC), and HAuNP@DTTC nanoparticles in DI water. **Fig. S2**. In vitro photoacoustic imaging of HAuNP@DTTC as PA contrast agents. (a) Combined ultrasound and photoacoustic images of PA phantom images under 808 nm excited laser for HAuNP@DTTC diepersion with different Au concentrations (from 0 to 20 OD). (b) Average PA signal as a function of wavelength and nanoplatforms concentration. **Fig. S3**. SERS spectra of Hollow Au-coupled DTTC (HAuNP@DTTC) nanoplatform in PBS. **Fig. S4**. Cell viabilities of 4T1 tumor cells incubated with HAuNP@DTTC with different concentrations for 24 h. **Fig. S5**. TEM micrographs of normal tissue (left) and tumor tissue (right). The red arrows denoted the HAuNP@DTTC. **Fig. S6**. Anti-tumor effect in vivo. Photographs of the 4T1-bearing mice at different time points after the various treatment. **Fig. S7**. Photographs of mice blood incubated with HAuNP@DTTC (1.6-200 μg mL -1) and corresponding percent hemolysis; water and PBS as the positive and negative controls, respectively. **Table S1**. The tumor growth inhibition (TGI) values of the 4T1-bearing mice of different groups.

## Data Availability

All data generated or analyzed during this study are included in this manuscript and its supplementary material.
