# Supplementary material for: Low-dose X-ray enhanced tumor accumulation of theranostic nanoparticles for high-performance bimodal imaging-guided photothermal therapy
Source: J Nanobiotechnology. 2021 May 26;19:155. doi: 10.1186/s12951-021-00875-8 (PMC8152352; doi:10.1186/s12951-021-00875-8)
Supplement: Supplementary file 1 — Additional file 1: Fig. S1. (a) Hydrodynamic size changes of HAuNP@DTTC nanoparticles in different physiological solutions (H2O, PBS, FBS, DMEM + 10 % FBS) for 7 days; (b) Corresponding photographs of the first and seventh day; (c) Zeta potential of hollow Au, hollow Au with DTTC complex (Hollow Au/DTTC), and HAuNP@DTTC nanoparticles in DI water. Fig. S2. In vitro photoacoustic imaging of HAuNP@DTTC as PA contrast agents. (a) Combined ultrasound and photoacoustic images of PA phantom images under 808 nm excited laser for HAuNP@DTTC diepersion with different Au concentrations (from 0 to 20 OD). (b) Average PA signal as a function of wavelength and nanoplatforms concentration. Fig. S3. SERS spectra of Hollow Au-coupled DTTC (HAuNP@DTTC) nanoplatform in PBS. Fig. S4. Cell viabilities of 4T1 tumor cells incubated with HAuNP@DTTC with different concentrations for 24 h. Fig. S5. TEM micrographs of normal tissue (left) and tumor tissue (right). The red arrows denoted the HAuNP@DTTC. Fig. S6. Anti-tumor effect in vivo. Photographs of the 4T1-bearing mice at different time points after the various treatment. Fig. S7. Photographs of mice blood incubated with HAuNP@DTTC (1.6-200 μg mL -1) and corresponding percent hemolysis; water and PBS as the positive and negative controls, respectively. Table S1. The tumor growth inhibition (TGI) values of the 4T1-bearing mice of different groups. [file 12951_2021_875_MOESM1_ESM.raman]

Supporting Information

Low-dose X-ray enhanced tumor accumulation of theranostic nanoparticles for high-performance bimodal imaging-guided photothermal therapy

Qiaolin Wei^1,2,5*^, Jian He^2*^, Shuaifei Wang^3*^, Shiyuan Hua^2^, Yuchen Qi ^2^, Fangyuan Li^3^, Daishun Ling^3，6#^ and Min Zhou^1,2,4#^

1. Eye Center, The Second Affiliated Hospital, Zhejiang University School of Medicine, Hangzhou 310009, China

2. Institute of Translational Medicine, Zhejiang University, Hangzhou, 310029, China

3. Institute of Pharmaceutics, College of Pharmaceutical Sciences, Zhejiang University, Hangzhou, 310058, China.

4. State Key Laboratory of Modern Optical Instrumentations, Zhejiang University, Hangzhou, 310058, China

5. Institute of Pharmacy, School of Medicine, Hangzhou Normal University, Hangzhou, 311121, China

6. Frontiers Science Center for Transformative Molecules, School of Chemistry and Chemical Engineering, National Center of Translational Medicine, Shanghai Jiao Tong University, Shanghai 200240, China

These authors contributed equally to this work.

^#^ **Address correspondence to**

zhoum@zju.edu.cn (Min Zhou);

dsling@sjtu.edu.cn (Daishun Ling)

**Materials.**

All chemicals and reagents were used as received without further purification. HAuCl_4_·3H_2_O (≥99.9% trace metal basis), AgNO_3_ were obtained from Sinopharm Chemical Reagent Co., Ltd. (Shanghai, China). Raman reporter 3,3’-diethylthiatricarbocyanine iodide (DTTC; 99%) was purchased from Sigma-Aldrich (St. Louis, MO, USA). De-ionized water (18.2 MΩ·cm-1) was prepared by a Milli-Q purification system (St. Louis, MO, USA) and used in all experiments. Dulbecco's modified eagle's medium (DMEM), fetal bovine serum (FBS) and trypsin-EDTA were obtained from Gibco-BRL (Burlington, ON, Canada).

**Experiment Methods.**

**Characterization of HAuNP@DTTC.**

Structural morphology and element mapping of hollow Au NP-coupled DTTC (HAuNP@DTTC) were investigated using JEM-2100 field-emission transmission electron microscopy (TEM) (FETEM, JEOL, Inc. Japan). X-ray diffraction patterns (XRD) were characterized by a D8 Advance (Bruker, Germany). Hydrodynamic size and zeta potential were measured using a Malvern Zetasizer Nano-ZS90 (Malvern, UK). Ultraviolet-visible near-infrared (UV-vis-NIR) spectra were recorded with a Shimadzu 2600 UV-vis-NIR spectrophotometer (Shimadzu, Kyoto, Japan). The Renishaw InVia microscopy system equipped with a 785-nm laser (Renishaw Inc., U.K.) was used for the collecting of SERS spectra and images.

**Cell viability.**

Cell viability was evaluated using the 3-(4,5-dimethylthiazol-2-yl)-2,5-diphenyltetrazolium bromide (MTT) assay kit (YEASEN, Shanghai, China). Live/dead cells were stained using a Calcein-AM/PI double stain kit (YEASEN, Shanghai, China), and imaged under a fluorescence microscope (Zeiss, Oberkochen, Germany). Mouse breast carcinoma 4T1 cells were cultured in DMEM medium (10% FBS, 1% antibiotics) at 37 °C and under a humidified atmosphere containing 5% CO_2_. 4T1 cells were seeded in a 96-well plate at 5×10^3^ cells per well for 24 h, then the HAuNP@DTTC was incubated with the cells for another 24h. For the photothermal therapy (PTT) group, the 4T1 cells were exposed to an 808 nm laser (MDL-N-808-10W, Changchun, China) at 1.5 W cm^-2^ for 3 min. Pre-irradiation (Pre-IR) was performed under 3 Gy of X-ray (X-RAD 160, PXi, USA). For the Pre-IR+PTT group, cells were exposed to 3 Gy X-ray irradiation, incubated with NPs for 4 hours, treated with an 808 nm laser 1.5 W cm^-2^ for 3 min and then rested for another 4 hours before MTT assay. The optical density (OD) at 490 nm wavelength of each well was recorded on a SpectraMax M5 plate reader (Molecular Devices, San Jose, CA, USA). All experiments were repeated three times.

**Animal models.**

In all *in vivo* experiments, female Balb/c-nude mice that were 5 weeks old at the start of the experiment were used. For the orthotopic 4T1 breast tumors model, 2 × 10^6^ 4T1 cells were implanted into the thoracic mammary fat pads of the mice. The animals were euthanized once tumors of the control group reached a size of more than 10 mm in diameter. After animal euthanization, tumor tissues and major organs (heart, liver, spleen, lung, and kidney) were excised, washed with PBS, and fixed in 4% formaldehyde. 4T1 cell line was purchased from the American Type Culture Collection (ATCC), and the cell culture was under the manufacturer’s guidelines. All animal research was performed by the guidelines from the Institutional Animal Care and Use Committee of Zhejiang University School of Medicine.

***In vivo* biosafety analysis.**

Toxicity experiments were carried out with 6-week old male Balb/c mice. Mice were injected through the tail vein with hollow HAuNP@DTTC (100 μg mL^-1^; 200 μL/mouse) or an equal volume of PBS (n =5~6 per group). Body weight of the mice was recorded every two days until the mice were sacrificed fourteen days later. For the hematologic and clinical chemistry analysis, the mice blood was obtained through the cardiac puncture, then the organs of the mice were fixed in 4% PFA for pathology analysis.

**Immunohistochemistry.**

The mice of all the five groups were sacrificed 14 days post-treatment, the tumors and major organs (heart, liver, spleen, lung, and kidney) were excised and fixed using 4% PFA for 24 h, and then embedded in paraffin for immunohistological staining. The PFA fixed sections were first stored up to 2 days at room temperature and then sectioned into 5 µm thick slices, mounted on glass slides, deparaffinized, and rehydrated for staining. From each tumor, 4 sections were stained with hematoxylin and eosin (H&E), Ki-67, HSP-70 and cluster of differentiation 31 (CD31). After then, the sections were imaged using virtual slide microscopy (Olympus VS120, Olympus Life Sciences, Waltham, MA, USA). Staining with H&E was used for general surveying of tumor morphological alterations following therapy and was not quantified. Staining with Ki-67, HSP-70 and CD31 were quantified by computing a ratio of the corresponding stained areas to the whole tumor cross-sectional area. Endothelial-specific CD31 staining was used to quantify microvascular densities (MVD) and to complement the increased accumulation of NPs in tumor regions. This was done by counting the number of stained blood vessels in 4-5 fields of view (vessels per view) under a 20× objective lens.


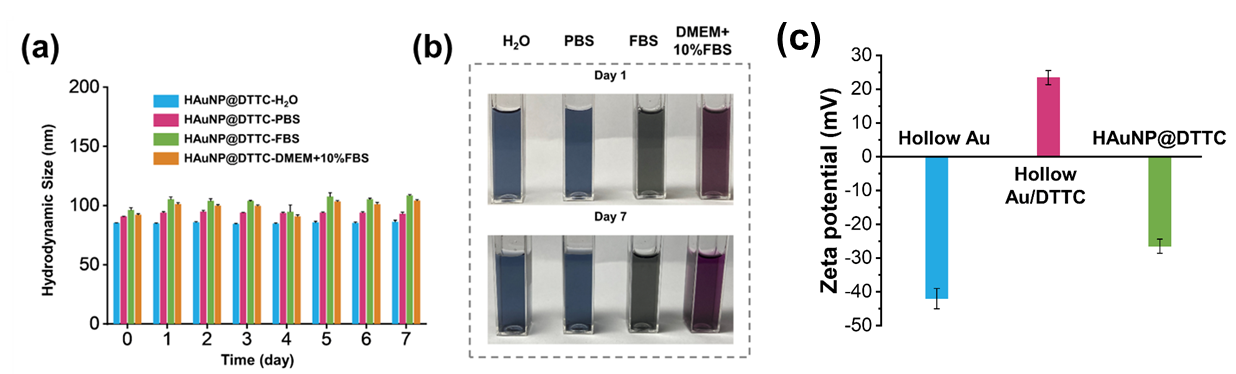


**Fig. S1.** (a) Hydrodynamic size changes of HAuNP@DTTC nanoparticles in different physiological solutions (H_2_O, PBS, FBS, DMEM + 10 % FBS) for 7 days; (b) Corresponding photographs of the first and seventh day; (c) Zeta potential of hollow Au, hollow Au with DTTC complex (Hollow Au/DTTC), and HAuNP@DTTC nanoparticles in DI water.


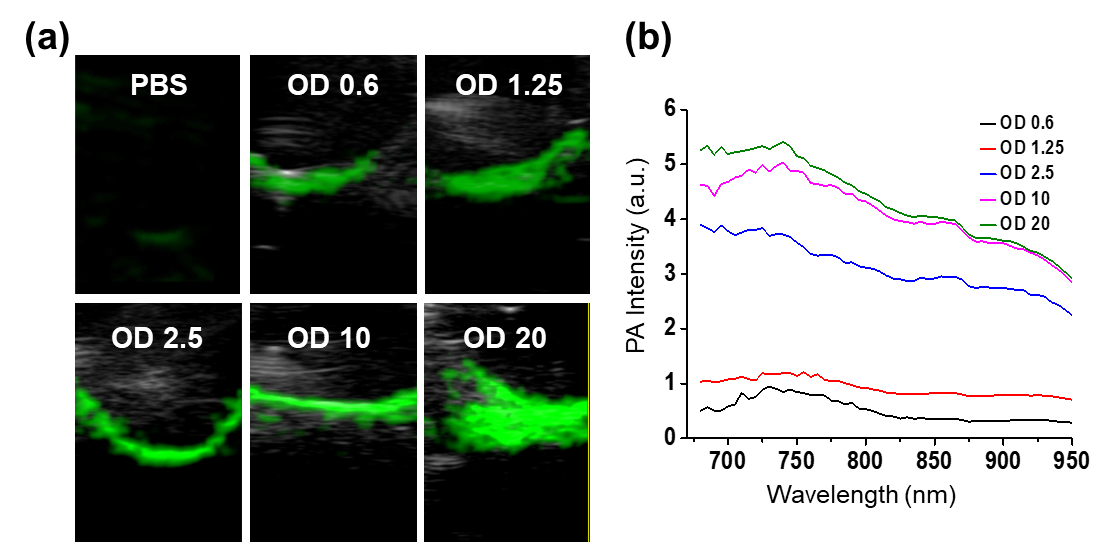


**Fig. S2.** *In vitro* photoacoustic imaging of HAuNP@DTTC as PA contrast agents. (a) Combined ultrasound and photoacoustic images of PA phantom images under 808 nm excited laser for HAuNP@DTTC dispersion with different Au concentrations (from 0 to 20 OD). (b) Average PA signal as a function of wavelength and nanoplatforms concentration.


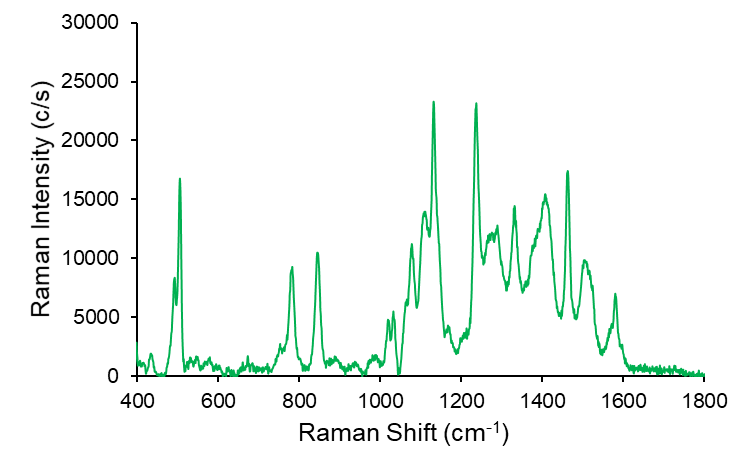


**Fig. S3.** SERS spectra of Hollow Au-coupled DTTC (HAuNP@DTTC) nanoplatform in PBS.


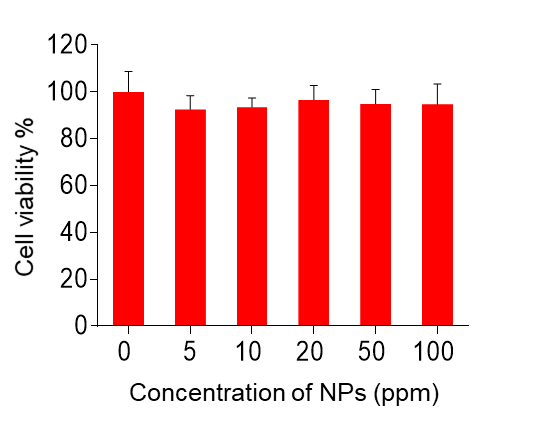


**Fig. S4.** Cell viabilities of 4T1 tumor cells incubated with HAuNP@DTTC with different concentrations for 24 h.


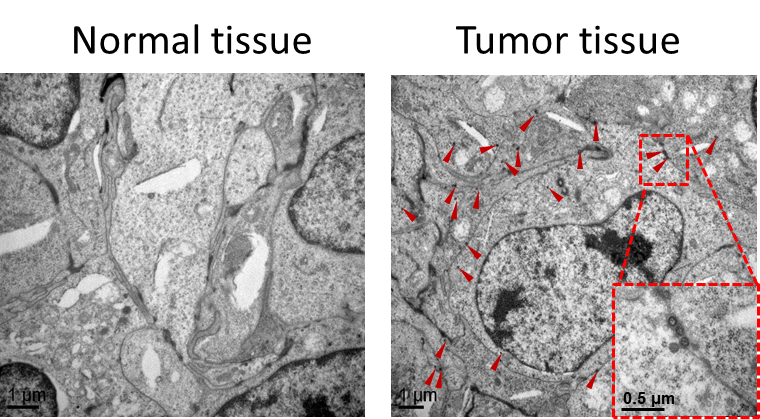


**Fig. S5.** TEM micrographs of normal tissue (left) and tumor tissue (right). The red arrows denoted the HAuNP@DTTC.


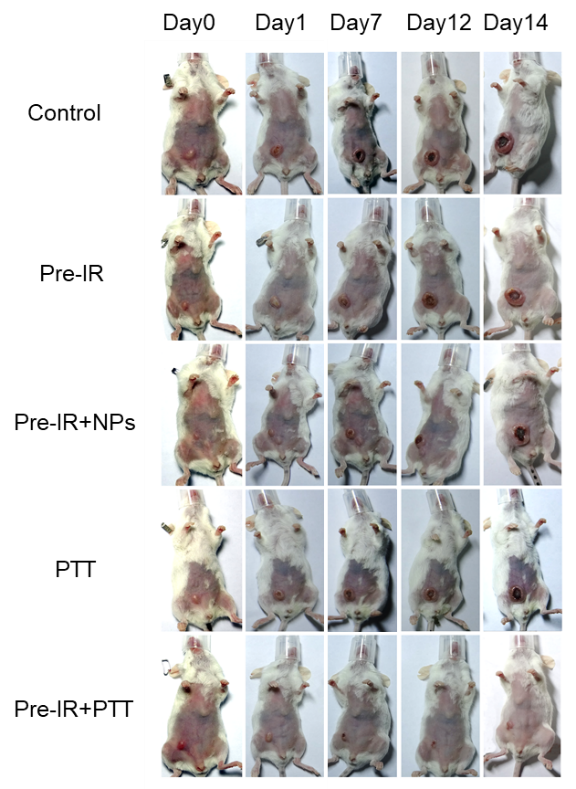


**Fig. S6.** Anti-tumor effect *in vivo*. Photographs of the 4T1-bearing mice at different time points after the various treatment.


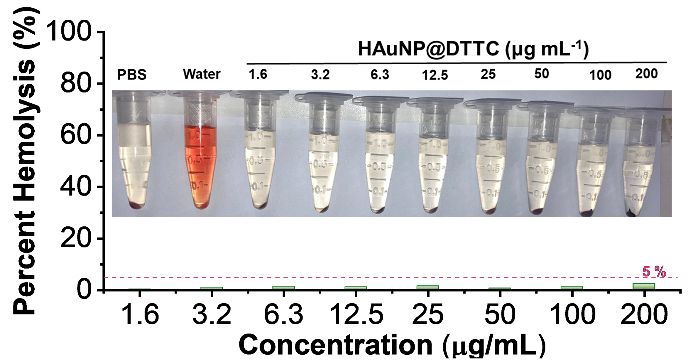


**Fig. S7.** Photographs of mice blood incubated with HAuNP@DTTC (1.6-200 μg mL ^-1^) and corresponding percent hemolysis; water and PBS as the positive and negative control group, respectively.

**Table 1.** The tumor growth inhibition (TGI) values of the 4T1-bearing mice of different groups.

**
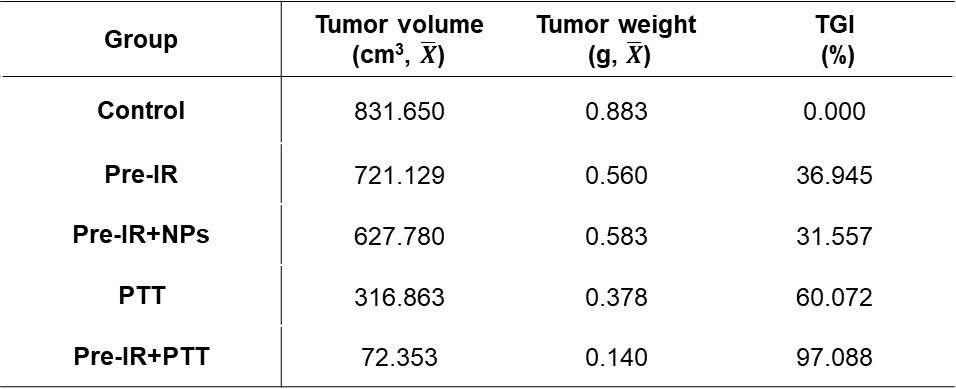
**
